# Interlaboratory Comparison as a Source of Information for the Product Evaluation Process. Case Study of Ceramic Tiles Adhesives

**DOI:** 10.3390/ma15010253

**Published:** 2021-12-29

**Authors:** Cristina Stancu, Jacek Michalak

**Affiliations:** 1 Ceprocim S.A., 6, Blvd. Preciziei, Sector 6, 062203 Bucharest, Romania; cristina.stancu@ceprocim.ro; 2 Research and Development Center, Atlas sp. z o.o., 2, Kilińskiego St., 91-421 Lodz, Poland

**Keywords:** interlaboratory comparison (ILC), proficiency testing (PT), risk analysis, measurements uncertainty (MU), ceramic tiles adhesive (CTA), assessment and verification of constancy of performance (AVCP), construction product, market surveillance

## Abstract

In this study, the results obtained by 19 laboratories participating in 2 editions of the interlaboratory comparison (ILC) determining 2 properties of ceramic tiles adhesives (CTAs), i.e., initial tensile adhesion strength and tensile adhesion strength after water immersion following EN 12004, were analyzed. The results show that participating laboratories maintain a constant quality of their work. The use of z-score analysis, under ISO 13528, allows for classifying 89.5% to 100% of laboratories as satisfactory, depending on the measurement’s kind and edition. The remaining laboratories are classified as questionable. The investigation of the predominant mode of failure of the CTA’s samples tested in the two editions shows significant differences. From the perspective of laboratories, the goal of the ILC has been achieved. From the standpoint of a manufacturer who evaluates a product’s properties when placing it on the market, the results indicate the necessity of a particular treatment of the product evaluation process because the variability of the obtained results is significant. It increases the possibility of the product failing to meet the assessment criteria verified by the construction market supervision authorities. The manufacturer must consider all possible variations in the risk analysis, including the ILC results, to improve the assessment process of CTAs.

## 1. Introduction

The assessment and verification of constancy of performance (AVCP) of products in EU countries is a complex and multidimensional process. The rules for placing construction products on the market define the Construction Products Regulation (CPR) [1]. This document describes a harmonized system of assessing, performance expression, and conditions for CE marking, while controlling the constancy of the assessment results, which should remain constant. 

The laying down of a product on the market is always associated with a risk, which is considered in different categories. Risk is an ambiguous concept, challenging to define. However, risk assessment is a fundamental technical framework for systematically analyzing the risk associated with an industrial activity [2]. Effective risk management requires essential knowledge about people’s perceptions of risk in their industry [3]. The construction industry, in general, is risky, and the risks involved in building construction objects are highly complex [4]. The risk related to the quality of construction products is only one out of several dozen identified in the construction process [5]. In the scientific literature, the risk related to the construction product’s non-compliant evaluation criteria is often not presented [6,7,8,9,10], very rarely from the manufacturer’s perspective [11,12]. 

It is essential for all market participants that the products are safe. Knowledge from internal and external sources should be considered to create any product. In most cases, in-house knowledge is dominant, and, of course, producers know the level of safety/risk related to their product development. From the manufacturer’s perspective, it is a problem when market supervision bodies will negatively assess their product. A particular situation is when this happens due to negative test results ordered by market surveillance authorities, and, of course, it may occur in many cases. One of them is when the actual values of the product’s performance are close to the threshold value of the evaluation criterion (declared value), and when the evaluation methods are incorrectly selected. A proper, holistic understanding of risk allows manufacturers to make appropriate decisions to protect against adverse effects, including uncertainty analysis [6,7,13]. Such understanding is crucial to avoid contentious situations. Conducting a risk analysis by the product’s manufacturer, including the measurement uncertainty (MU) related to the measured and declared performance of the product, is fundamental for the correct determination of the stability of the product’s performance and safe use. It is also necessary for the continuous maintenance of the product as compliant with the assessment criteria. All measurements are erroneous; it is essential to know what size of measurement error accompanies the measurement [14]. However, since it is not possible to identify the sources and values of all systematic errors (and their directions) for any given measurement result, as well as the value of the random error occurring at a given time, the MU criterion is commonly used, inter alia, in clinical chemistry [15,16,17]. This parameter characterizes the dispersion of the quantity values attributed to a measurand (quantity intended to be measured) based on the information used [18]. It is essential to add that MU is a “non-negative” parameter [14]. Failure by the manufacturer to consider the consequences of uncertainty, including MU, during product development and the production process may result in a situation in which the product assessed as conforming may be non-compliant. It is also possible that a product rejected as non-compliant may be a compliant product [19,20]. Note that uncertainty in measurements is an operational concept that only relates to quantitative values assigned to the measurand based on the available information, the model of the measurement procedure, and the probabilistic assumptions used [21]. However, it is also essential to be aware that the measurement information provides only partial information about the actual product characteristic. When taken into account during the product assessment, it minimizes the adverse effects of incorrect evaluation. Still, it is not always sufficient to permanently deliver a product that complies with the assessment criteria [22]. Knowing the MU value for a given measurement method minimizes the risk of obtaining results that do not meet the evaluation criterion; for example, modifying the product recipe resulting in a change (usually increase) of the product parameter value. Such an operation, however, is generally associated with an increase in production costs. Of course, the MU related to the testing method of product properties is one of the many factors that the manufacturer needs to consider.

All laboratories aim to provide reliable information. Most of the measurements aim to assess compliance with a specification or regulation [23]. In this case, the measurement itself is not the goal but the basis for making objective decisions [24]. Conformity decisions are made for different products in many application areas without transparent and harmonized risk sharing, due to MU between the consumer and the producer/supplier of the product [24]. Performing measurements to assess compliance with specifications requires further development. Mainly, where shortcomings are observed, i.e., in the description of the measurement process, recognition that confidence in the measured result depends not only on its uncertainty but also on its integrity and further development of validated methods for the performance assessment [25,26]. Interlaboratory research plays an essential role in ensuring the quality of laboratory testing. The growing interest in this topic reflects the scientific literature [9,27,28,29,30,31,32,33,34,35,36]. In interlaboratory comparisons (ILCs) dominate clinical chemistry, biochemistry, medicine, and pharmacy researches. Construction products are much less the subject of the ILCs. In addition, the testing and assessment of construction products is still not precisely defined in terms of uncertainty, methods of estimation, and taking into account [8]. For construction products, a small number of tests and a limited number of participants are ILC limitations [9,36]. An additional difficulty is that most of the measurement methods used for assessment with evaluation criteria are destructive in the case of construction products. Thus, it is not possible to repeat the test with the same sample. In the future, the situation will change due to the standardization regulations creating new requirements for selected construction products related to the possibility of ILC/PT performance [37]. 

ILC is considered primarily in the aspect of proficiency testing (PT), understood as part of a quality system that provides an external assessment that a laboratory’s performance meets the requirements. PT is also understood to mean that an individual laboratory evaluates its performance for the intended purposes. A significant tenet of ILC is to prove the laboratory’s ability to reproduce the results generated by the other laboratories. The ILC is also considered a learning exercise and is associated with terms such as quality control and certification. Those, as mentioned above, are the main goals of ILCs. In the scientific literature, ILCs are not perceived as a tool providing information to the manufacturer that can be used to verify the product recipe, so that the product meets the evaluation criteria during external evaluation. ILCs are also not considered a tool to verify the test methods specified in the standards for product assessment.

This study compares the results of the ILCs of ceramic tile adhesives (CTAs) organized by Ceprocim, carried out in two editions, i.e., in 2019–2020 and 2020–2021. The research organized by Ceprocim aimed to demonstrate that the systematic participation of laboratories in ILC improves the quality of their work. Based on the ILC results, the importance of the laboratories’ participation in the PT will be analyzed. The authors will analyze the obtained results from the perspective of laboratories participating in the ILC. The ILC results will also be considered in the manufacturer’s risk analysis, accompanying the product evaluation process. Based on the results, the authors will discuss the potential need to modify the requirements and methodology specified in EN 12004 [38]. There arises the need for discussion due to the application by market surveillance authorities of the simple acceptance rule, which does not consider the variability resulting from measurement uncertainty. 

As mentioned before, few scientific papers discuss ILC for construction materials. Additionally, the articles published so far consider the ILC/PT in terms of assessing the competence of the work of laboratories. Conclusions resulting from PT are discussed between laboratories and institutions that granted laboratory accreditation. There are no studies in the scientific literature discussing how the results obtained in the ILC/PT can be the subject of a risk analysis conducted by the producer. Results of the ILC/PT also indicate possible modifications to the standards to improve them and make them more useful for both producers and users of products.

## 2. Materials and Methods

### 2.1. Short History of Interlaboratory Comparisons Organized by Ceprocim

In 2007, the Romanian laboratory Ceprocim (notified body number 1830), authorized to test in the scope of EN 12004, initiated a project of ILC of the initial adhesion strength of cementitious CTAs. Nine laboratories, mainly Romanian, participated in the first edition of ILC, while twenty-seven laboratories of research institutes and manufacturers of CTAs from the following nine countries participated in the fifth edition: Austria, Bulgaria, Croatia, the Czech Republic, Germany, Poland, Portugal, Romania, and Slovenia [30]. Proficiency tests/interlaboratory comparisons organized by Ceprocim were carried out according to uniform rules and the requirements of EN ISO/IEC 17043 [39]. All laboratories used identical concrete slabs for the tests and the same ceramic tiles provided by the test organizer. According to the study’s authors, more than 90% of the test results obtained by the participating laboratories can be described as satisfactory (|z| ≤ 2) according to EN ISO/IEC 17043, and the remaining were questionable or unsatisfactory [30].

In 2014, Ceprocim extended the research to the second characteristic—adhesion strength after water immersion. In 2018, during the tenth jubilee edition of the study, three characteristics were measured: initial adhesion strength, adhesion strength after immersion in water, and open time [34]. The last extension of the scheme took place in 2020, when two more tests were introduced: tensile adhesion strength after heat aging and tensile adhesion strength after the freeze-thaw cycle [40]. Randomly assigning a code number to each laboratory in each edition ensures confidence in the entire study. Reference to each laboratory in all reports is made by code number. 

Concrete slabs of various origins were used for the twelve editions of the ILC. Each of the laboratories participating in the eleventh and twelfth editions used their own concrete slabs.

It is also important to note that the laboratories participating in ILCs organized by Ceprocim represent both accredited laboratories according to EN ISO/IEC 17025 [41] and non-accredited laboratories. Twenty-nine laboratories participated in the eleventh edition of the ILC (2019–2020) and twenty-seven in the twelfth edition a year later. Nineteen laboratories participated in both the eleventh and twelfth editions, and the results obtained by these laboratories are discussed later in this paper. The 19 laboratories are from the following countries: Austria—1, Germany—3, Greece—2, Italy—1, Mauritius—1, Poland—1, Portugal—1, Republic of Moldova—1, Romania—5, Slovenia—1, Spain—1, and United Arab Emirates—1.

### 2.2. Ceramic Tile Adhesives (CTAs)

CTAs are an important group of construction products intended to install ceramic cladding for internal and external purposes [42]. Ceramic tiles are commonly used on all continents. In 2020, their production reached 16.093 billion m^2^, while consumption was slightly lower and amounted to 16.035 billion m^2^ [43]. Assuming an average consumption of 4 kg CTA per 1 m^2^ of ceramic cladding, this means a global production of about 65 million tons of CTAs. 

Requirements for all CTAs (cementitious, dispersion, and reaction resin) applying to all member states of the EU, three of the EFTA members (Iceland, Norway, and Switzerland), and other states (United Kingdom, North Macedonia, Serbia, and Turkey) specify EN 12004. EN 12004 was first published in 2001, and the last version of the standard published in the list of European harmonized standards [44] is EN 12004:2007+A1:2012 [38]. The next version of the standard published by CEN in 2017, i.e., EN 12004-1:2017 [45], has not yet been included in the list of harmonized standards published in the Official Journal of the European Union and, therefore, cannot be the basis for the assessment and verification of constancy of performance. Based on the EN 12004, the global standard ISO 13007-1 was implemented in 2004 [46]. Establishing the standard specifying requirements, terminology, working methods, and application properties for CTAs for internal and external tile installations on walls and floors by the ISO organization has resulted in their harmonization worldwide. The current ISO 13007-1 standard comes from 2014 [47].

The fundamental issue in ILC is that the tested product (CTA), and other materials used for the tests, i.e., concrete slabs and ceramic tiles, are identical. The organizer provided the CTA and ceramic tiles in the analyzed studies, and each of the participating laboratories provided concrete slabs. Residual CTA determinations were repeatedly performed on a 250 μm sieve to ensure that the CTA was homogeneous. Checking the adhesive homogeneity was performed with the same equipment, by the same operator, during a short period. The sample was considered homogeneous when all the results had been placed in the range: average residue value on the 250 μm sieve ± 2 s (%). The value of s represents the standard deviation of repeatability.

The CTA used in the research was classified as C2E under the requirements of EN 12004 [38]. The initial adhesion strength and adhesion strength after water immersion were determined following the test methods and using auxiliary materials (concrete slab, ceramic tiles) specified in EN 12004 [38]. Finally, it is essential to note that each ILC participant received also written guidelines, in addition to the CTA, for the study and ceramic tiles.

### 2.3. Evaluation of the Results Using the z-Score

For the statistical calculation, algorithm A in Annex C from the standard ISO 13528 [48] was used. It implies, for initial adhesion strength, for tensile adhesion strength after heat aging, for tensile adhesion strength after water immersion, for tensile adhesion strength after freeze-thaw cycle, and for open-time after not less than 30 min, calculation of the robust values for average and for standard deviation, from the results obtained of each participant.

Based on an iterative calculation, the calculus of robust average (x*) and the robust standard deviation (s*) were made. The calculation was carried until there was no change from one iteration to the next in the third significant figure in the robust standard deviation and the equivalent figure in the robust average. The value obtained for the robust average after the last iteration represents the assigned value (*x_pt_*), chosen to be the consensus value.

The standard uncertainty *u*(*x_pt_*) of the assigned value is given by Equation (1):

*u*(*x_pt_*) = 1.25 × *σ_pt/_*√*p* (1)

where:

*σ_pt_*—standard deviation for proficiency assessment,
*p*—the number of participant laboratories that carried on the test on concrete slab.

The z-score is calculated with Equation (2):

*_Zi_* = *x_i_* − *x_pt_*/σ*_pt_* (2)

where:

*x_i_*—the value obtained by each participant for each test,
*x_pt_*—the assigned value on total participants for each test.

The evaluation of the results was made according to EN ISO/IEC 17043:2010, as follows:

- satisfactory, when |z| ≤ 2;
- questionable, when 2 < |z| < 3;
- unsatisfactory, when |z| ≥ 3.

In the z-score calculation program, the assigned value and the robust standard deviation value obtained after the last iteration have been used as they result from calculation without being round. 

The interpretation mentioned above of z-score is conventional (see ISO/IEC 17043:2010 [30], B.4.1.1.). A result that gives 2.0 < |z| < 3.0 is considered to give a warning signal. Participants of the ILC/PT should be advised to check their measurement procedures following warning signals if they indicate an emerging or recurrent problem. The justification for using the limits of 2.0 and 3.0 for z-score is as follows. Measurements that are carried out correctly are assumed to generate results that can be described (after transformation, if necessary) by a normal distribution with mean x_pt_ and standard deviation σ_pt_. z-score, which will then be normally distributed with a mean of zero and a standard deviation of 1.0. Under these circumstances, only about 0.3% of scores would be expected to fall outside the range −3.0 ≤ z ≤ 3.0, and only about 5% would be expected to fall outside the range −2.0 ≤ z ≤ 2.0.

## 3. Results

The initial tensile adhesion strength, tensile adhesion strength after water immersion of CTA, and the predominant mode of failure obtained in the eleventh edition of the ILC (2019–2020) and the twelfth edition (2020–2021) are presented in Table 1 and Table 2.

It is worth adding that two other failure patterns not listed in Table 1 and Table 2 are possible, namely, CF-S - cohesive failure in the substrate or CF-T - cohesive failure in the tile.

## 4. Discussion

### 4.1. ILC Results in the Light of ISO 13528 Guidelines

Table 3 shows the lowest and highest values of the initial tensile adhesion strength and tensile adhesion strength after water immersion of CTA reported by the laboratories participating in the ILC, out of 19 laboratories participating in both the eleventh and twelfth editions, 29 laboratories participating in the eleventh edition (2019–2020), and 27 laboratories participating in the twelfth edition (2020–2021). 

The guidelines specified in ISO 13528 [48], including the recommendations on the interpretation of proficiency testing data, were applied to analyze the results obtained in the ILC. Table 4 shows the results of the calculations made following ISO 13258 [48]. 

**Figure 1:**
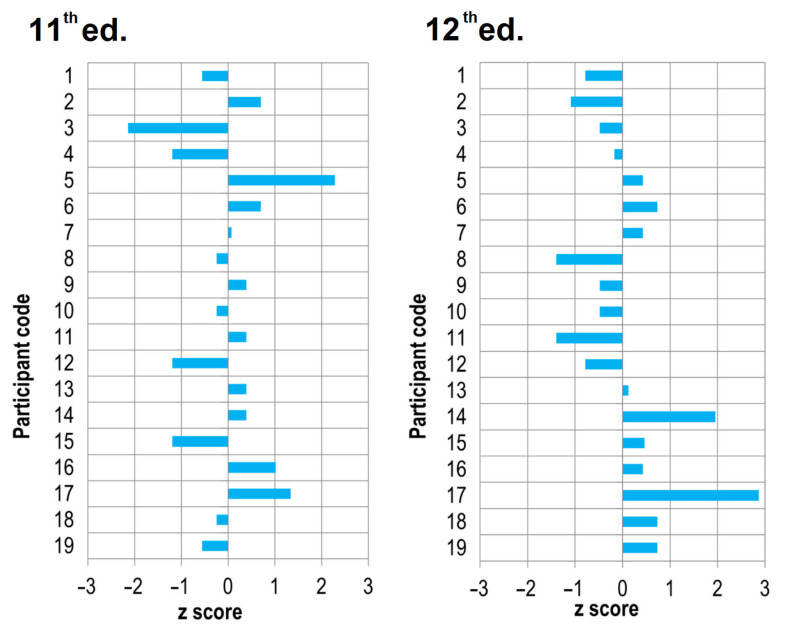
The z-score value for the initial tensile adhesion strength for each of 19 laboratories participating in the eleventh and twelfth editions of the ILC.

**Figure 2:**
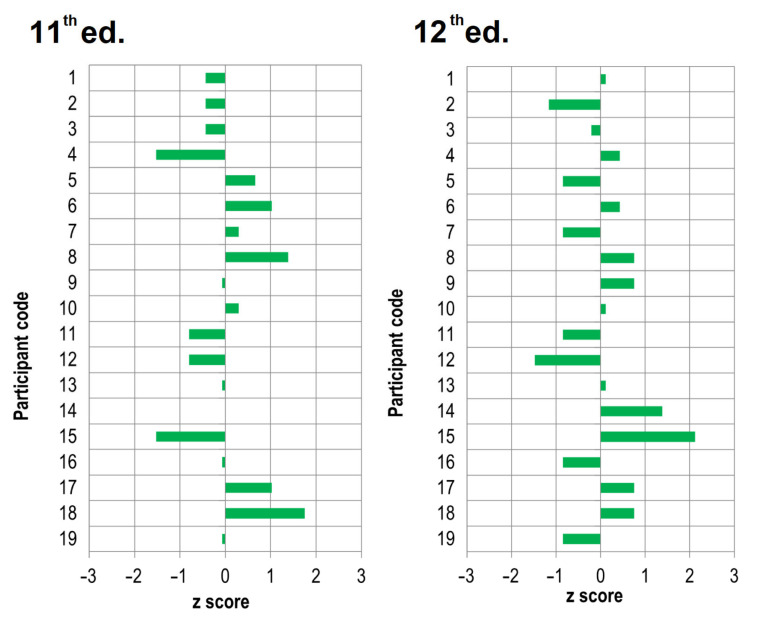
The z-score value for the measurement of tensile adhesion strength after water immersion for each of 19 laboratories participating in the eleventh and twelfth editions of the ILC.

The z-score values calculated following the Equation (2) for each laboratory for the initial tensile adhesion strength and tensile adhesion strength after water immersion measurements are presented in Figure 1 and Figure 2, respectively. 

The analysis of the z-score shows that in the eleventh ed., 2 out of 19 laboratories in the scope of the initial tensile adhesion strength measurement obtained results classified as “questionable” (2 < |z| < 3), and the remaining 17 laboratories obtained the “satisfactory” status (|z| ≤ 2). A year later, in the twelfth ed. of the ILC, only 1 laboratory was labeled “questionable” based on the results obtained, while the remaining 18 laboratories were “satisfactory”. In the case of the tensile adhesion strength after water immersion measurement in the eleventh ed. of the ILC, the results obtained by all laboratories allowed them to receive the status of “satisfactory”, and in the following twelfth ed. of the ILC, 1 of the 19 laboratories received the result referred to as “questionable”. The z-score analysis also allows for the indication among of the 19 laboratories participating in both editions, those whose obtained measurement results are the closest assigned value (consensus value), namely laboratories marked with numbers: 1, 7, 9, 10, and 13 and, in further order, 19, 6, 16, and 18. When analyzing the obtained results, it is worth noting that among the results for which the z-score is |z| ≤ 2, these are the results classified as “satisfactory” from the perspective of the measurement laboratory and the analysis of ILC results under the requirements of ISO 13528 [48]. Another dimension of this result is from the product evaluation perspective. Although the z-score meets the condition of |z|≤ 2, for example, in the case of the initial tensile adhesion strength measurement in the eleventh ed., it means both 1.2 N/mm^2^ or 1.3 N/mm^2^ (participant code 4, 12, and 15), next to 2.0 N/mm^2^ or 2.1 N/mm^2^ (laboratories marked as 16 or 17). There is a significant difference between the value of 1.3 and 2.1, which may cause the product to be assessed as failing the acceptance criterion when it is not. The situation can become even more difficult for the product and its manufacturer when the product is reassessed by a market surveillance authority external to the manufacturer. The difficulty level may increase when market surveillance authorities apply the simple acceptance rule that does not consider the variability resulting from MU. Although the obtained results are classified as “satisfactory” in the ILC/PT evaluation categories, differences between the results are significant from the product evaluation perspective, and the product manufacturer cannot ignore this fact in their product evaluation. Producers after the so-called “safe side” must also consider the measurement variability in the value of the acceptance criterion. As shown by the results of ILC studies, this value is significant for CTAs tested under EN 12004 requirements. The analysis of the results of the predominant mode of failure showed more significant differentiation between laboratories. The obtained results are summarized in Table 5.

**Figure 3:**
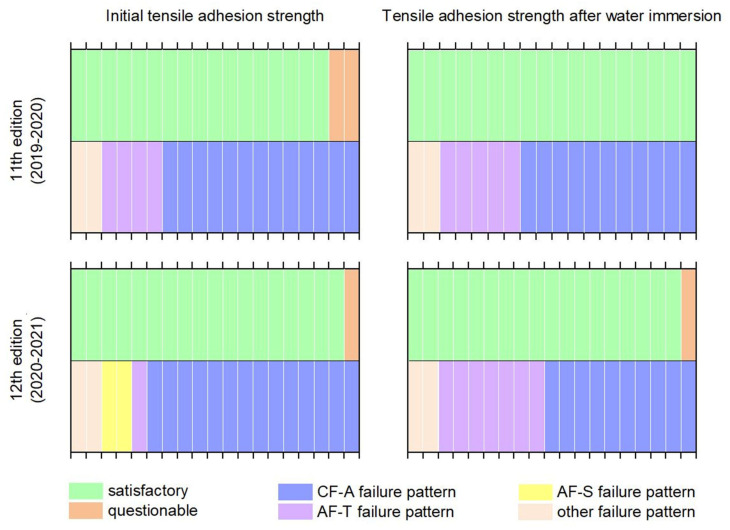
Summary of the results of the CTA measurements in two editions of the ILC.

Figure 3 shows a schematic summary of the results of the CTA studies in two subsequent editions of the ILCs. The data presented in Figure 3 combines the data previously included in Figure 1 and Figure 2, plus Table 5. Figure 3 shows no correlation between the laboratory classification using the z-score and the observed failure pattern of tensile adhesion strength, regardless of whether the subject of the measurement was the initial or after immersion in water tensile adhesion strength. The lack of this correlation, and a different distribution of the observed mode of predominant failure, is an additional argument that the interpretation of the initial tensile adhesion strength and tensile adhesion strength after immersion in water results should be approached with caution.

One of the goals of the Romanian Ceprocim project was to show that constant participation in the laboratory proficiency testing programs improves the quality of laboratories work. In this respect, the organizers of the study achieved the intended goal. Analyzing the results of both editions of the ILC/PT with the participation of the same 19 laboratories, it can be concluded that the differences between the individual editions are minor, but they do exist. It leads to the conclusion that laboratories maintain a constant level of the quality of the performed measurements. In addition, this is one dimension of the ILC/PT being conducted.

### 4.2. Factors Influencing the Measurement of Tensile Adhesion Strength

From a practical point of view (in risk analysis), the reproducibility of the results, i.e., the degree of agreement between the results obtained by different analysts in different laboratories using a given measurement procedure, is essential for the producers of CTAs. In this regard, while discussing the results of the PT described in this paper, one should also pay attention to several other aspects. 

Felixberger [49] described the results of initial adhesion tests of 7 cementitious CTAs, performed in 10 laboratories using 2 different concrete slabs. As the first concrete slab, each of the laboratories participating in the study used a standard concrete slab meeting the requirements of EN 1323:2007 [50]. The second concrete slab was purchased by the research organizer and delivered to all participating laboratories. The standard deviation of the measurement described by Felixberger ranged from 15% to 20%, showing also the influence of concrete slab on the value of the determined adhesion. Felixberger found that for cementitious CTAs with a lower adhesion value, more significant differences between individual measurements exist than in the case of adhesives with higher adhesion. Felixberger formulating the conclusions, stating that the priming of the concrete slab surface for testing would result in the uniformity of the slab surface in terms of its absorption properties. It also creates a situation closer to the actual use where manufacturers of cementitious CTAs recommend using a primer before laying ceramic tiles [49].

It is worth noting here that the subject of the analysis in this study was C2E classified CTA, i.e., with higher adhesion strength values. In the case of the research results analyzed in this article, the concrete slab effect was also present. 

Another factor worth noting is the water used to season the samples. The EN 12004 standard does not contain any requirements in this respect. When testing the effect of seasoning water on the adhesion of CTAs, samples were stored in three types of water: in distilled water (pH = 7.09, specific conductivity = 0.040 mS/cm), in tap water (pH = 8.25, specific conductivity equal 0.805 mS/cm), and in softened water (pH = 8.63, specific conductivity equal 1.228 mS/cm) [51]. The tests showed that the origin and type of water used to season the samples significantly affects the adhesion of CTAs. Samples stored in distilled water represent a higher adhesion value than in the two other cases. The differences between the test results were so significant that they decided the fulfillment of the evaluation criteria.

The adhesion of CTAs is determined in the following system: concrete substrate (slab)—CTA—ceramic tile. Some properties of ceramic tiles approved for use during adhesion tests are specified in the standards referred to for a given test method in EN 12004. Niziurska assessed the influence of ceramic tiles’ chemical composition and surface structure on the adhesion of CTAs to tiles [52]. The results obtained in the tests confirmed the impact of the quality of auxiliary materials (ceramic tiles) used in the tests on the compliance of CTAs with evaluation criteria.

It is also worth noting that the result of the adhesion measurement (regardless of whether the initial tensile adhesion strength or the tensile adhesion strength after water immersion) is the result of two other measures. It means the maximum load of the sample during the tensile strength measurement and the sample surface (adhesion surface). Therefore, the accuracy of the destructive force determination and the accuracy of the sample cross-sectional area determination influence the accuracy of the final result (adhesion).

Considering the obtained results and additional conditions related to the tensile adhesion strength of CTAs measurements described above, the manufacturer designing the product during the risk analysis determining the value of the acceptance criteria has to consider the simultaneous occurrence of all possible variations that may accompany the measurement.

## 5. Conclusions

The analysis of the CTAs adhesion results performed by 19 laboratories participating in 2 consecutive ILC/PT editions showed that the ILC/PT objective was achieved. Laboratories participating in two successive editions maintain a similar level of quality of performed measurements. In the case of the initial adhesion strength measurement in the eleventh edition of the ILC, 89.5% of laboratories obtained the result “satisfactory” under the ISO 13528 criteria. A year later, the result was slightly better—94.7% of laboratories received a satisfactory result. Regarding the measurement of tensile adhesion strength after water immersion, all 19 laboratories were classified as “satisfactory” in the eleventh edition. A year later, one of the laboratories obtained results classified as “questionable”. The analysis of the results in the field of the predominant mode of failure showed more significant differences. The goal has been achieved from the perspective of the purpose of the ILC/PF. However, the results obtained from the perspective of the manufacturer whose product is being assessed by the supervisory authorities are not satisfactory. The differences between the results obtained between individual laboratories are significant.

The manufacturer of the product, knowing the acceptance criteria and being aware of the “imperfections” of the measurement method, must take all of that into account in their risk analysis. They must add to the value of the acceptance criterion all the possible variations, including the MU resulting from the measurement method. In their risk analysis, they must assume a variant in which all potential volatilities coincide. The ILC/PT results analyzed in this study indicate that the measurement method, with its imperfections, is an integral part of the final value of the acceptance criterion. In a situation where, when assessing a product by market surveillance authorities, the simple acceptance rule is applied, which does not take into account the variability resulting from the MU, the risk analysis must additionally take this circumstance into account.

The obtained ILC/PT results, although they are satisfactory in terms of the requirements for this type when assessing the competence of measurement laboratories, indicate significant imperfections of the measurement method. At the same time, the obtained results signal to the product manufacturer that they must pay special attention when determining the constancy of performance of their product. Otherwise, it can happen that their product will not meet the acceptance criteria (threshold value).

The results obtained in ILC/PT also indicate that the authors of EN 12004 may analyze possible revision of the standard. In the light of the results described in this study, it seems that it would be reasonable to introduce the obligatory requirement to determine the measurement uncertainty by providing the tensile adhesion strength result in EN 12004. Moreover, the provision that due to the specificity of the tensile adhesion strength measurements, it seems inappropriate for the assessment of CTAs application of the simple acceptance rule.

## Figures and Tables

**Table 1:** The initial tensile adhesion strength and tensile adhesion strength after water immersion of CTA with the predominant mode of failure obtained by 19 laboratories in the eleventh ed. (2019–2020).

Participant Code	Initial Tensile Adhesion Strength		Tensile Adhesion Strength after Water Immersion	
	[N/mm^2^]	Dominant Failure Pattern	[N/mm^2^]	Dominant Failure Pattern
1	1.5	AF-T	0.7	AF-T
2	1.9	70% CF-A/30% AF-T	0.7	50% CF-A/50% AF-T
3	1.0	CF-A	0.7	CF-A
4	1.3	AF-T	0.4	AF-T
5	2.4	CF-A	1.0	CF-A
6	1.9	CF-A	1.1	AF-T
7	1.7	CF-A	0.9	CF-A
8	1.6	CF-A	1.2	CF-A
9	1.8	CF-A	0.8	CF-A
10	1.6	CF-A	0.9	CF-A
11	1.8	AF-T	0.6	AF-T
12	1.3	CF-A	0.6	CF-A
13	1.8	CF-A	0.8	CF-A
14	1.8	CF-A		
15	1.3	CF-A	0.4	CF-A
16	2.0	70% CF-A/30% AF-T	0.8	40% CF-A/60% AF-T
17	2.1	CF-A	1.1	CF-A
18	1.6	AF-T	1.3	AF-T
19	1.5	CF-A	0.8	CF-A

CF-A—cohesive failure within the adhesive, AF-T—adhesion failure between adhesive and tile.

**Table 2:** The initial tensile adhesion strength and tensile adhesion strength after water immersion of CTA with the predominant mode of failure obtained by 19 laboratories in the twelfth ed. (2020–2021).

Participant Code	Initial Tensile Adhesion Strength		Tensile Adhesion Strength after Water Immersion	
	[N/mm^2^]	Dominant Failure Pattern	[N/mm^2^]	Dominant Failure Patten
1	1.5	CF-A	0.9	AF-T
2	1.4	50% CF-A/50% AF-T	0.5	5% CF-A/95% AF-T
3	1.6	CF-A	0.8	CF-A
4	1.7	CF-A	1.0	CF-A
5	1.9	CF-A	0.6	AF-T
6	2.0	CF-A	1.0	AF-T
7	1.9	CF-A	0.6	CF-A
8	1.3	CF-A	1.1	CF-A
9	1.6	CF-A	1.1	CF-A
10	1.6	CF-A	0.9	AF-T
11	1.3	AF-S	0.6	AF-T
12	1.5	AF-T	0.4	AF-T
13	1.8	CF-A	0.9	CF-A
14	2.4	AF-S	1.3	AF-T
15	1.9	CF-A	1.5	CF-A
16	1.9	50% CF-A/50% AF-T	0.6	20% CF-A/80% AF-T
17	2.7	CF-A	1.1	CF-A
18	2.0	CF-A	1.1	CF-A
19	2.0	CF-A	0.6	CF-A

CF-A—cohesive failure within the adhesive, AF-T—adhesion failure between adhesive and tile, AF-S—adhesion failure between adhesive and substrate.

**Table 3:** The lowest and highest values of the initial tensile adhesion strength and tensile adhesion strength after water immersion of CTA obtained by 19 laboratories and by all participating laboratories in the eleventh and twelfth editions.

ILC Edition	No. of Laboratories	Initial Tensile Adhesion Strength [N/mm^2^]		Tensile Adhesion Strength after Water Immersion [N/mm^2^]	
		Lowest Value	Highest Value	Lowest Value	Highest Value
The same laboratories participating in both ILC editions					
11th (2019–2020)	19	1.0	2.4	0.4	1.3
12th (2020–2021)	19 *	1.3	2.4	0.4	1.5
All participating laboratories					
11th (2019–2020)	29	0.3	2.6	0.4	1.9
12th (2020–2021)	27	1.3	2.7	0.4	2.0

* Eighteen laboratories reported results for the measurements of the tensile adhesion strength after water immersion.

**Table 4:** The value of statistical parameters calculated following ISO 13258 [48] for measurements of CTA initial tensile adhesion strength and tensile adhesion strength after water immersion during the eleventh and twelfth editions of the ILC.

Parameter	Initial Tensile Adhesion Strength		Tensile Adhesion Strength after Water Immersion	
	11th ed.	12th ed. *	11th ed.	12th ed.
x* [N/mm^2^]	1.7	1.8	0.8	0.9
s* [N/mm^2^]	0.3	0.3	0.2	0.3
*x_pt_* [N/mm^2^]	1.7	1.8	0.8	0.9
σ*_pt_* [N/mm^2^]	0.3	0.3	0.3	0.3
*u*(*x_pt_*)	0.1	0.1	0.1	0.1
*V*	18.9	18.6	33.5	36.2

x*—robust average of the results reported by all participating laboratories; s*—robust standard deviation of the results reported by all laboratories; *x_pt_*—assigned value—consensus value; σ*_pt_*—standard deviation for proficiency assessment; *u*(*x_pt_*)—standard uncertainty of the assigned value; *V*—coefficient of variation. * Eighteen laboratories reported results for the measurements of the tensile adhesion strength after water immersion.

**Table 5:** The number of the predominant mode of failure for the initial tensile adhesion strength and tensile adhesion strength after water immersion measurements in the eleventh and twelfth ed. of the ILC.

Predominant Failure Pattern	Initial Adhesion		Adhesion after Water Immersion	
	11th ed.	12th ed. *	11th ed.	12th ed.
AF-S	0	2	0	0
AF-T	4	1	5	7
CF-A	13	14	11	10
Other	2	2	2	2
Total	19	19	18	19

* Eighteen laboratories reported results for the measurements of the tensile adhesion strength after water immersion in the eleventh edition (2019–2020).

## Data Availability

The data presented in this study are available upon request from the corresponding author.
